# An update on pharyngeal assessment by the modified barium swallow

**DOI:** 10.1007/s00261-024-04707-9

**Published:** 2024-12-08

**Authors:** Jessica Zarzour, Jonathan Revels, Brinda Rao Korivi, Bonnie Martin-Harris

**Affiliations:** 1 University of Alabama at Birmingham, Birmingham, USA; 2 New York University, New York, USA; 3 The University of Texas MD Anderson Cancer Center, Houston, USA; 4 Northwestern University, Evanston, USA

**Keywords:** Modified barium swallow, Dysphagia, Swallowing

## Abstract

The modified barium swallow study (MBSS) is a diagnostic examination that visualizes the functional anatomy and physiology of the oral pharyngeal swallowing mechanism in real time. The MBSS, a videofluoroscopic imaging method, is indicated for patients with known or suspected oropharyngeal dysphagia and ideally involves the combined expertise of a radiologist and speech pathologist. The MBSS provides critical diagnostic insights that help in identifying and assessing the type and severity of physiological swallowing impairments, evaluating the safety of oral intake, testing the effectiveness of evidence-based interventions, and developing treatment plans. This manuscript aims to present an overview of MBSS standards from an interdisciplinary perspective, emphasizes key areas of best practices, and reviews the common morphologic abnormalities seen on MBSS exams.

**Supplementary Information:**

The online version contains supplementary material available at 10.1007/s00261-024-04707-9.

## Introduction

The modified barium swallow study (MBSS) is a diagnostic examination that visualizes the functional anatomy and physiology of the oral pharyngeal swallowing mechanism in real time. The MBSS, a videofluoroscopic imaging method, is indicated for patients with known or suspected oropharyngeal dysphagia (difficulty swallowing) and ideally involves the combined expertise of a radiologist and speech pathologist. The study provides critical diagnostic insights that help in identifying and assessing the type and severity of physiological swallowing impairments, evaluating the safety of oral intake, testing the effectiveness of evidence-based interventions, and developing treatment plans [1–6]. The precise diagnostic data obtained from MBSS, when combined with clinical evaluations, patient histories, and clinician expertise, form the foundation for diagnosing physiological swallowing disorders and identifying targets for behavioral interventions aimed at improving swallowing function [3–9].

MBSS should utilize a standardized, reliable, and validated protocol for capturing and reviewing videofluoroscopic images, which is considered a best practice [4]. The technical aspects of the examination, such as fluoroscopy settings, recording and playback devices, should be standardized to ensure diagnostic accuracy. Empirical evidence supports the use of continuous fluoroscopic settings (at 30 pulses per second) to accurately capture swallowing movements and airway protection mechanisms relative to bolus flow as they occur in real time [4, 6, 10–12]. Additionally, it is crucial to conduct a risk/benefit analysis related to patient safety, severity of aspiration, clinical yield, and radiation exposure (As Low As Reasonably Achievable, ALARA) for both patients and clinicians when determining the necessity and approach of the MBSS [13, 14]. Recent data show that implementation of validated standards keeps radiation exposure to a minimum average of 2.9 min, with a 95% confidence interval of 2.8–3.0 min, and 0.27 mSv per MBSS [15–18].

MBSS is applicable not only in inpatient settings but also in rehabilitation, long-term care, home health, and outpatient environments. The collaboration between radiologists and speech-language pathologists (SLPs) is essential for optimizing the performance of MBSS and providing the best care for patients. This manuscript aims to present an overview of MBSS standards from an interdisciplinary perspective, emphasizing three key areas of best practices: acquiring essential diagnostic information, adhering to patient and clinician safety recommendations, and maintaining technical standards and resource utilization.

## Standardized protocol

The MBSS is designed to offer a comprehensive examination of the swallowing mechanism rather than focusing solely on feeding. The main objectives are to identify and evaluate the presence, type, and severity of physiological swallowing impairments, determine the safety and efficiency of oral intake, assess the impact of frontline interventions, and develop targeted therapeutic and nutritional management plans [1, 3, 8, 19].

A standardized protocol (Table 1), characterized by validated core elements or standards, ensures transparency, reproducibility, accurate and reliable measurements, and clear expectations regarding the procedure and outcomes. Flexibility within these core elements allows for reasonable adjustments based on the patient’s clinical circumstances or specific clinical questions [6]. Barium sulfate suspension is the contrast agent used in boluses of varying consistencies to visualize liquids and foods similar to those on a patient’s meal tray [20].

**Table 1:** Modified barium swallow study impairment profile (MBSImP) standardized protocol

	Lateral view
TRIAL 1	
Bolus Type	Thin Liquid (40% w/v, 4 CPS)
Volume	Spoon (5 mL)
Instruction	“Hold this in your mouth until I ask you to swallow.” Then, “Swallow when you’re ready.”
TRIAL 2	
Bolus Type	Thin Liquid (40% w/v, 4 CPS)
Volume	Spoon (5 mL)
Instruction	“Hold this in your mouth until I ask you to swallow.” Then, “Swallow when you’re ready.”
TRIAL 3	
Bolus Type	Thin Liquid (40% w/v, 4 CPS)
Volume	Cup sip (20 mL)
Instruction	“Take a sip as you normally would but hold it in your mouth until I ask you to swallow.” Then, “Swallow when you’re ready.”
TRIAL 4	
Bolus Type	Thin Liquid (40% w/v, 4 CPS)
Volume	Sequential swallow (40 mL)
Instruction	“Drink this in your usual manner until I tell you to stop.”
TRIAL 5	
Bolus Type	Nectar-Thick Liquid (40% w/v, 300 CPS)
Volume	Spoon (5 mL)
Instruction	“Hold this in your mouth until I ask you to swallow.” Then, “Swallow when you’re ready.”
TRIAL 6	
Bolus Type	Nectar-Thick Liquid (40% w/v, 300 CPS)
Volume	Cup sip (20 mL)
Instruction	“Take a sip as you normally would but hold it in your mouth until I ask you to swallow.” Then, “Swallow when you’re ready.”
TRIAL 7	
Bolus Type	Nectar-Thick Liquid (40% w/v, 300 CPS)
Volume	Sequential swallow (40 mL)
Instruction	“Drink this in your usual manner until I tell you to stop.”
TRIAL 8	
Bolus Type	Honey-Thick Liquid (40% w/v, 1500 CPS)
Volume	Spoon (5 mL)
Instruction	“Hold this in your mouth until I ask you to swallow.” Then, “Swallow when you’re ready.”
TRIAL 9	
Bolus Type	Pudding (40% w/v)
Volume	Spoon (5 mL)
Instruction	“Swallow when you’re ready.”
TRIAL 10	
Bolus Type	Cookie
Volume	1/2 Cookie with 3 mL Pudding (40% w/v)
Instruction	“Chew this as you normally would and swallow when you feel ready.”

	A/P view
TRIAL 11	
Bolus Type	Nectar-Thick Liquid (40% w/v, 300 CPS)
Volume	Spoon (5 mL)
Instruction	“Hold this in your mouth until I ask you to swallow.” Then, “Swallow when you’re ready.”
TRIAL 12	
Bolus Type	Pudding (40% w/v)
Volume	Spoon (5 mL)
Instruction	“Swallow when you’re ready.”

Using factory-produced standardized barium sulfate preparations minimizes the risks associated with aspiration of food and liquid materials and ensures compliance with safety regulations. These preparations are aligned with levels on the International Dysphagia Diet Standardization Initiative (IDDSI) and are used in developing standardized, validated measures of swallowing physiology [7, 21–24].

Both the SLP and radiologist should be skilled in identifying physiological elements of swallowing impairment within the context of multiple patient factors. This expertise helps predict how patients will swallow with subtle changes in consistency or other modifications impacting bolus flow without the necessity of attempting to replicate all food consistencies in a fluoroscopy suite. This practice introduces infection control and aspiration risks and prevents comparison of findings on follow-up evaluations. Because MBSS provides a brief sampling of swallowing function, findings should be confirmed by the SLP through direct or consultative observation of patient performance at the bedside or during mealtime [8].

Metrics for quantifying the type and severity of swallow impairment involve surrogate visuoperceptual measures of muscle contraction, pressure generation, airway protection, and efficiency of clearance of swallowed material [25–27]. Based on the multidimensional swallowing function, multiple scales may be applied depending on the clinical or research question of interest.

**Fig. 1:**
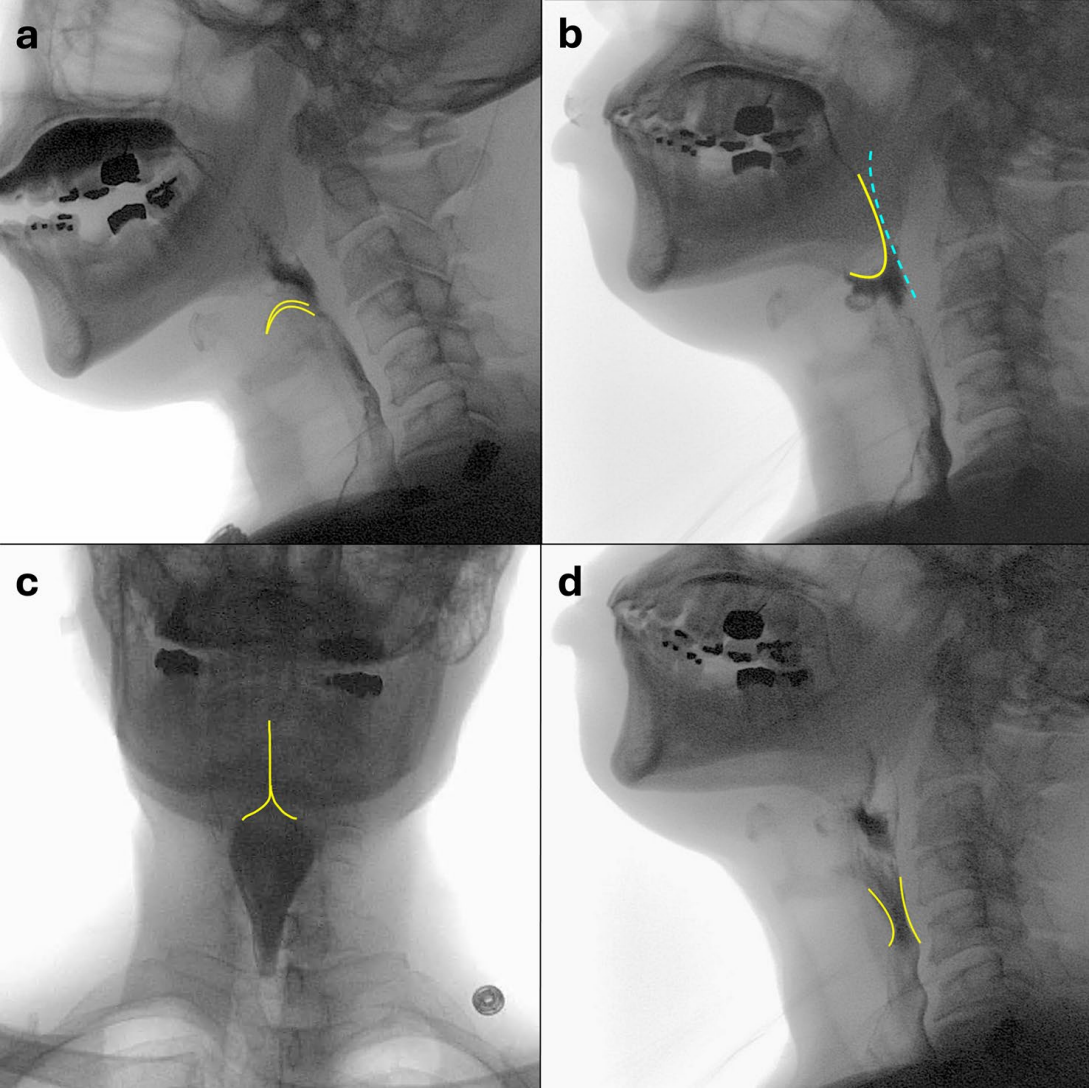
Select pharyngeal domain components including critical elements of airway protection such as laryngeal elevation and laryngeal vestibular closure (**a**), contraction of the tongue base and apposing pharyngeal constrictors (**b**), shortening and compression of the pharynx (**c**), and opening of the pharyngoesophageal sphincter (**d**)

Key components of swallowing physiology fall within three functional domains: oral, pharyngeal, and esophageal (Table 2) [24, 27]. The oral domain includes components related to oral control and tongue movement, including sensory input for initiation of pharyngeal swallowing. The components of pharyngeal domain include critical elements of airway protection such as laryngeal elevation and laryngeal vestibular closure (Fig. 1a), contraction of the tongue base and apposing pharyngeal constrictors (Fig. 1b), shortening and compression of the pharynx (Fig. 1c), and opening of the pharyngoesophageal sphincter (PES, Fig. 1d). The esophageal domain focuses only on the degree of esophageal bolus clearance in the upright position because of the evidence supporting the influence esophageal clearance on oropharyngeal swallowing behavior and therapy strategies [24, 28, 29]. Examination of esophageal function goes beyond the scope of the MBSS.

**Table 2:** MBSImP functional domains, physiologic components, and score ranges

Domain	Component	Score range
Oral	C1. Lip Closure C2. Tongue Control During Bolus Hold C3. Bolus Preparation/Mastication C4. Bolus Transport/Lingual Motion C5. Oral Residue C6. Initiation of the Pharyngeal Swallow	(0–4) (0–3) (0–3) (0–4) (0–4) (0–4)
Pharyngeal	C7. Soft Palate Elevation C8. Laryngeal Elevation C9. Anterior Hyoid Excursion C10. Epiglottic Movement C11. Laryngeal Vestibular Closure C12. Pharyngeal Stripping Wave C13. Pharyngeal Contraction C14. Pharyngoesophageal Segment Opening C15. Tongue Base Retraction C16. Pharyngeal Residue	(0–4) (0–3) (0–2) (0–2) (0–2) (0–2) (0–3) (0–3) (0–4) (0–4)
Esophageal	C17. Esophageal Clearance in the Upright Position	(0–4)

The Modified Barium Swallow Impairment Profile (MBSImP), developed by Martin-Harris and colleagues, is an essential tool for standardizing the assessment and reporting of MBSS (Table 1). The MBSImP identifies seventeen components of swallowing function and bolus clearance, providing a specific, consistent, accurate, and objective framework for interpreting and communicating swallowing impairments [3, 24]. This tool enhances the robustness of the MBSS by increasing inter-rater reliability and facilitating comparisons across different patient populations and clinical settings.

The MBSImP uses standardized barium sulfate preparations (VARIBAR® barium sulfate 40% weight/volume; Bracco Diagnostics, Inc., Monroe Township, NJ), which includes varied and validated consistencies (thin liquid, thin honey, honey, nectar, pudding) and swallowing tasks (teaspoon and cup administration) shown to influence swallowing physiology and impairment [21, 30, 31]. This standardization ensures that the same definitions and measurements are applied consistently, improving the quality of the assessment and supporting the development of tailored therapeutic regimens [8]. By employing the MBSImP, clinicians can better determine which strategies may improve swallowing function and safety, leading to more effective and personalized treatment plans.

The American College of Radiology (ACR) and the Society of Pediatric Radiology (SPR) have emphasized the importance of using standardized, validated protocols to reduce variability and ensure reproducibility in MBSS examinations. The 2023 update to the ACR-SPR Practice Parameter for MBSS underscores the clinical benefits of these standardized protocols and the use of commercially available barium sulfate products, which enhance diagnostic consistency and patient safety [4, 6].

The MBSS report should include quantitative, standardized measures of physiological swallowing impairment, the presence and response to penetration and aspiration, and the effect of compensatory strategies [8, 9, 24, 32]. Recommendations made by the SLP may also incorporate evidence-based exercises for improving the strength and skill of swallowing movements that consider pathophysiological reasoning and clinical experience [33].

## Morphological abnormalities

### Anatomy review

**Fig. 2:**
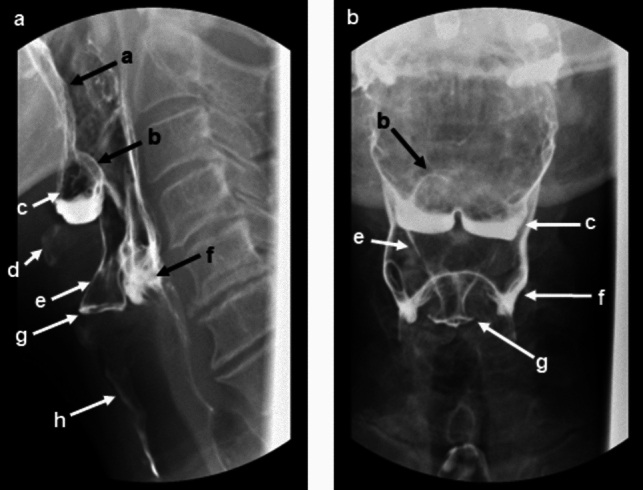
Lateral and AP images of the pharynx demonstrating typical anatomy encountered during a MBSS. a. Tongue base, b. Epiglottis, c. Vallecula containing a small amount of residual barium, d. Hyoid bone, e. Laryngeal vestibule coated in barium, consistent with laryngeal penetration, f. Piriform sinuses containing a small amount of residual barium, g. Vocal cords coated in barium, h. Barium coating the anterior aspect of the trachea, consistent with tracheal aspiration

The role of the radiologist during a MBSS is multifaceted and includes to be able to accurately detect functional and structural abnormalities. It is important for the radiologist to be familiarized with key anatomical landmarks of the pharynx (Fig. 2a, b). The pharynx is divided into the nasopharynx, oropharynx, and hypopharynx. The nasopharynx is bound inferiorly by the soft palate and the oropharynx is bound inferiorly by the hyoid bone or base of the epiglottis, with the pharyngoepiglottic fold is its true dividing landmark. The tongue base extends from the mouth to the valleculae. The epiglottis is superior and posterior to the valleculae. The hypopharynx includes the piriform sinuses which are the lateral boundary of the hypopharynx and extends to the cricoid cartilage [34, 35].

### Airway penetration

During the MBSS, the radiologist and SLP should assess for functional and structural abnormalities in the oral, pharyngeal, and esophageal phases of swallowing [24]. While the presence of laryngeal penetration and tracheal aspiration should be assessed, the cause for the airway penetration should also be determined. Airway penetration may occur prior to, during, or after the swallow. Laryngeal penetration occurs when contrast enters the laryngeal vestibule and tracheal aspiration occurs when contrast enters the trachea. A widely applied metric for grading the degree of airway penetration and the patient’s response is the Penetration-Aspiration Scale [32]. When aspiration or penetration are detected, the SLP may perform compensatory swallow strategies to minimize or improve the degrees of penetration or aspiration, including chin tuck, head turn, or breath-hold maneuvers.

It is anticipated that dysphagic patients may aspirate during a MBSS, as one of the primary objectives of the study is to identify and address the underlying causes of airway invasion. The customized barium contrast used in a standardized protocol is administered in small, controlled amounts. It is non-water soluble, thereby reducing the risk of chest infections. However, efforts should be made to minimize the volume of aspiration while maximizing the diagnostic value of the study. A key question that radiologists and speech pathologists face is: "How much aspiration is too much?"—a point at which the study may need to be discontinued. A patient's tolerance to aspiration varies depending on individual clinical circumstances. For instance, frail elderly patients with limited mobility and a non-productive cough may be at higher risk for developing chest infections due to chronic microaspiration. In contrast, more physically active patients, such as those recovering from head and neck cancer, who retain strong respiratory defenses, may tolerate routine aspiration during eating and drinking without progressing to aspiration pneumonia. Thus, there are no rigid stopping rules for MBS based solely on the amount of aspiration. Instead, a careful balance must be struck between the clinical condition of the patient and the sufficiency of the information obtained for accurate diagnosis and treatment planning. This decision is best made collaboratively by the examiners based on the individual patient’s clinical presentation.

### Structural abnormalities in the pharynx and cervical esophagus

While there is a wide range of functional abnormalities impacting pharyngeal swallowing, there are many structural abnormalities that may be encountered during an MBS exam.

**Fig. 3:**
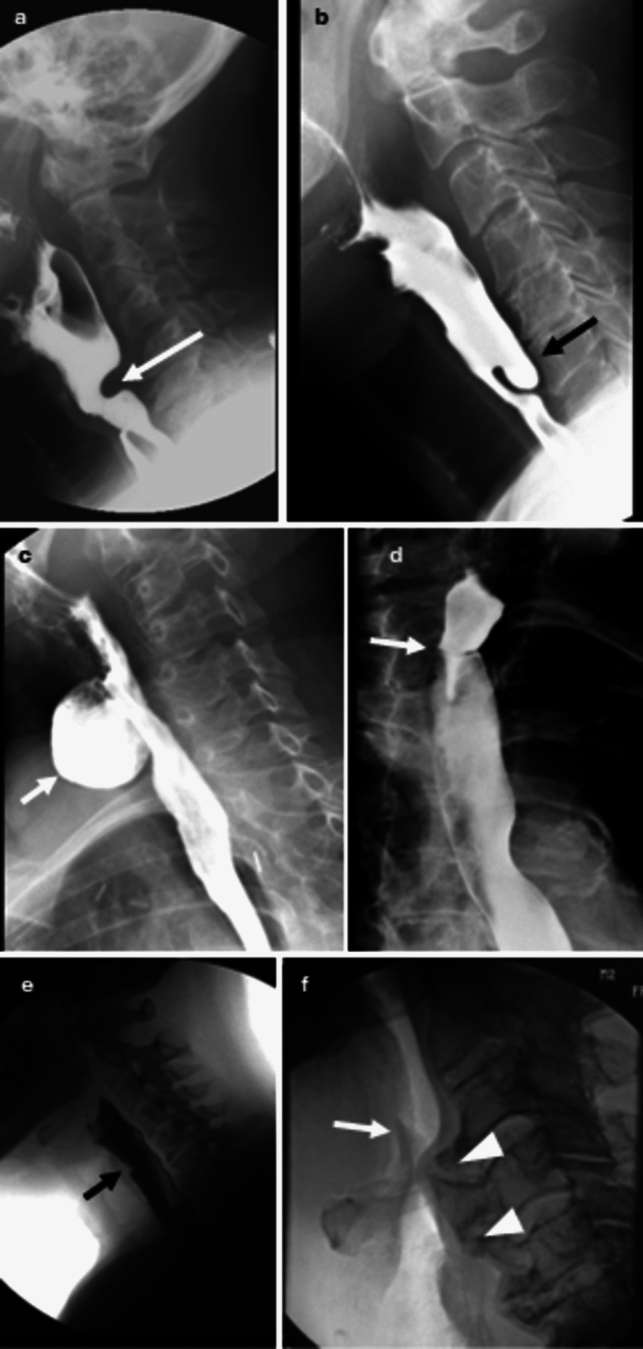
**a** Lateral image shows a cricopharyngeal bar (arrow) that narrows the cricopharyngeal junction by approximately 50%. **b** Lateral image shows a Zenker’s diverticulum (arrow) posterior and superior to the narrowed cricopharyngeus. **c** Oblique image shows a Killian–Jamieson diverticulum arising inferior to the cricopharyngeus and extending in an anterior and lateral fashion. **d** AP image demonstrates a thin cervical esophageal web (arrow). **e** Lateral image shows a post cricoid defect (arrow), a normal finding along the anterior aspect of the hypopharynx. **f** Large anterior cervical osteophytes (arrow heads) prevent inversion of the epiglottis (arrow)

The cricopharyngeal musculature is an important demarcation at the pharyngoesophageal junction and makes up the upper esophageal sphincter. It consists of the inferior constrictor muscle, the cricopharyngeal muscle, and circular fibers of the proximal cervical esophagus. The cricopharyngeus is closed between swallows and relaxes upon swallow initiation to allow the bolus to pass from the pharynx into the cervical esophagus [34]. A prominent posterior indention may be seen at the level of the cricopharyngeus and if mild, may be within the realm of normal or not clinically signification. However, if the cricopharyngeal “bar” results in significant luminal narrowing, it may result in dysphagia (Fig. 3a).

A Zenker’s diverticulum (Fig. 3b) is thought to form as a result of pulsion forces through an anatomic weak point (Killian’s dehiscence) at the pharyngoesophageal junction. This forms due to cricopharyngeal dysfunction which creates elevated intraluminal pressure [34, 36]. A Zenker’s diverticulum is located posteriorly or slightly to the left of midline and superior to the cricopharygeus. A Zenker’s diverticulum can be a source of food stasis that may cause multiple symptoms and complications, including dysphagia, halitosis, cough, and aspiration. Since the Zenker’s diverticulum is thought to arise from failure of the cricopharyngeus to relax, treatment is aimed surgical correction of the diverticulum along with a cricopharyngeal myotomy [37].

A Killian-Jameson diverticulum (Fig. 3c) protrudes through a muscular gap in the anterolateral wall of the proximal cervical esophagus, inferior to the cricopharyngeus [36]. Killian-Jameson diverticula are thought to form through this sidewall weakness, also known as the Killian-Jamieson space [35, 36]. Killian-Jameson diverticula are smaller and less common that Zenker’s diverticula [36]. Most are asymptomatic and rarely require treatment.

An esophageal web (Fig. 3d) is a thin membranous structure that can occur anywhere in the esophagus, but most commonly occurs in the cervical esophagus [38]. Most are considered idiopathic but can be seen in association with gastroesophageal reflux and Plummer-Vinson syndrome. The symptoms in patients can vary based on the degree of luminal narrowing caused by the web. On a barium swallow, a web appears as a thin (2–4 mm) transverse linear filling defect in the cervical esophagus along the anterior wall and often circumferentially. Cervical esophageal webs should not be mistaken for a normal post cricoid defect (image 3e). Post cricoid defects are caused by redundant mucosa or a submucosal venous plexus along the anterior wall of the hypopharynx that changes morphology upon swallowing [39, 40].

Anterior cervical osteophytes are common, and can occasionally be a cause of dysphagia predominantly through direct hypopharyngeal or esophageal compression, inflammation, or nerve impingement. Osteophytes/syndesmophytes in diffuse idiopathic skeletal hyperostosis (DISH) of the cervical spine may also result in pharyngeal dysphagia (DISHphagia) and may prevent inversion of the epiglottis which increases the risk for aspiration (Fig. 3f) [39]. Management of symptomatic cases typically involves conservative approaches such as anti-inflammatory and analgesic medications, postural adjustments, and dietary modifications. When these conservative measures prove insufficient, surgical resection of the osteophytes may be pursued, which has been shown to improve quality of life in some patients [41].

### Cervical soft tissue assessment

**Fig. 4:**
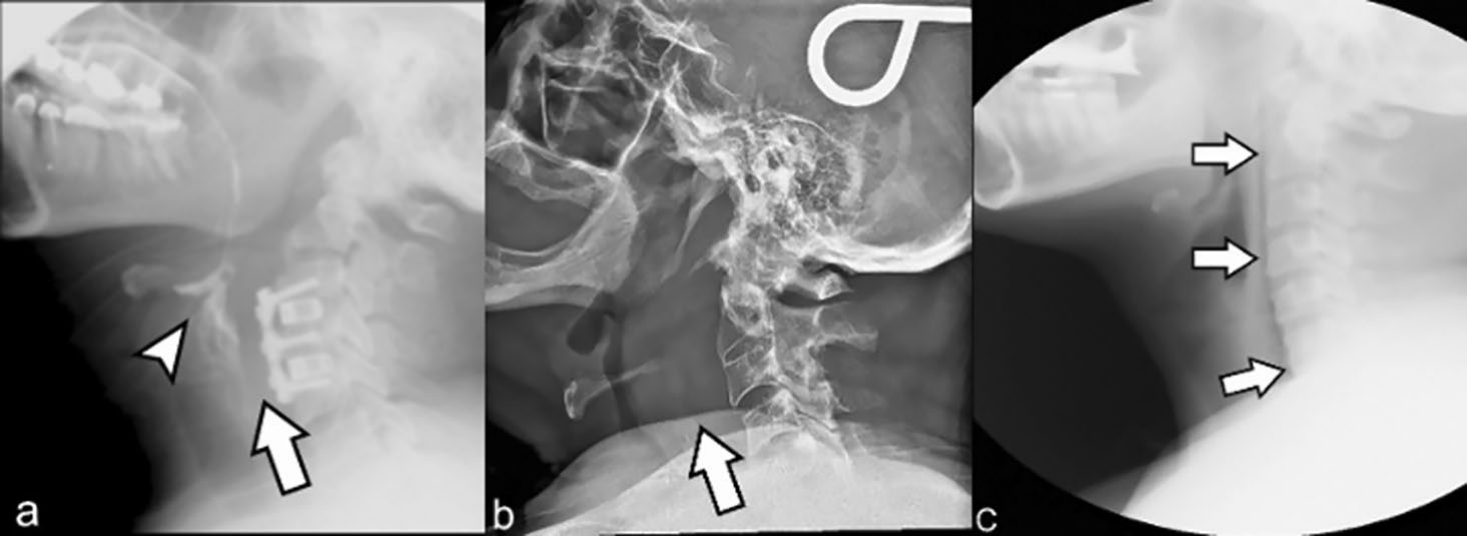
Abnormal prevertebral soft tissues on MBSS in 3 separate patients. **A** First patient, 1-day post-anterior cervical discectomy and fusion (ACDF) complaining of dysphagia. Prevertebral soft tissue thickening is present anterior to the fusion hardware (arrow). Laryngeal flash penetration is subtly present as well (arrowhead). On follow-up MBSS performed 6 days later (not shown), the soft tissue thickening had significantly decreased and no laryngeal penetration or aspiration was demonstrated. In the absence of an underlying fluid collection, prevertebral soft tissue swelling after ACDF will usually peak around 2–4 days and then gradually improves/decreases, resolves within 6 weeks. **B** Second patient complaining of neck pain and dysphagia, without recent surgical or trauma history. Prevertebral soft tissue thickening is present (arrow). Further clinical and imaging evaluation was recommended, which revealed discitis osteomyelitis due to methicillin-susceptible Staphylococcus aureus. **C** Third patient with history of asthma complaining of chest and neck pain, as well as dysphagia. Subtle linear lucency tracking along the prevertebral soft tissues (arrows), consistent with retropharyngeal emphysema. Chest radiograph demonstrated pneumomediastinum (not shown). Acknowledgement: Cases 2 and 3 are courtesy of Dr. Sarah Shaves, Eastern Virginia Medical School

Prevertebral soft tissue thickening (Fig. 4a and b) is a non-specific finding with a broad differential, including infection (such as retropharyngeal abscess), inflammation (e.g., longus colli calcific tendinitis), congenital conditions (like an abnormal retropharyngeal carotid artery course), as well as acute traumatic injuries and post-operative causes such as a hematoma and/or post-operative edema. Since this region is included in the field-of-view for MBSS studies, it should be routinely evaluated, as it may account for the etiology of the patient’s symptoms [42]. Amongst adults, the prevertebral soft tissue thickness at C2 should measure ≤ 7 mm or half of the vertebral body width, and at C7 it should be ≤ 22 mm or the width of the adjacent vertebral body [43]. Given the wide variety of causes for prevertebral soft tissue thickening, it is important to use correlative imaging studies to determine the potential etiology. In the absence of correlative imaging or relevant history (e.g., recent cervical spine fusion surgery), additional imaging may be warranted.

Similar to prevertebral soft tissue thickening, the presence of extraluminal gas (Fig. 4c) within the soft tissues of the head and neck observed during MBSS studies can result from various causes. These include head and neck infections, recent surgery, and conditions that originate in the thoracic region and ascend through the communicating fascial planes [44]. Identifying the etiology is crucial because some of these conditions can be life-threatening and may require immediate surgical intervention. Thus, careful evaluation and correlative imaging studies are essential for accurate diagnosis and timely management.

Cervical soft tissue densities can include normal calcifications associated with the aging process, evident in structures such as laryngeal cartilage and adjacent ligaments [43]. In contrast, certain densities may reflect pathological conditions, such as carotid artery calcifications or the presence of foreign bodies. Carotid calcifications can be indicative of an elevated stroke risk and may affect both treatment responses and patient prognosis. Although rare in adults, foreign body ingestion poses significant risks, potentially leading to severe complications like laryngeal and/or pharyngeal injuries, which can result in critical outcomes such as abscesses or mediastinitis. Consequently, precise diagnosis of foreign bodies is crucial, and should be included in the differential of cases of unexplained prevertebral soft tissue swelling and/or soft tissue gas [43, 45].

### Squamous cell carcinoma (SCC) of the larynx and complications

**Fig. 5:**
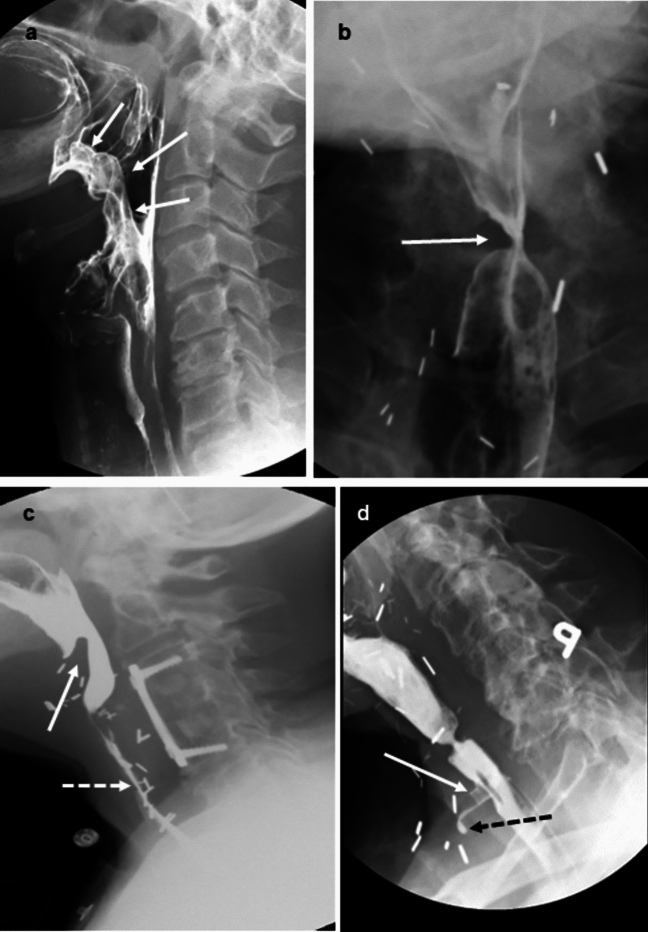
**a** Lateral image of a modified barium swallow demonstrates an irregular mass centered at the epiglottis and extending to the larynx which was later proven to be a squamous cell carcinoma (arrows). **b** This patient had a prior history of laryngeal squamous cell carcinoma and underwent total laryngectomy with free flap reconstruction. This AP image demonstrates an irregular stricture (arrow) along the right lateral aspect of the distal end of the neopharynx that was proven to be secondary to squamous cell carcinoma recurrence. **c** A band of scar tissue or “pseudoepiglottis” (arrow) at the tongue base after total laryngectomy. If the pseudoepiglottis impedes the flow of liquid and food, then it may lead to formation of a diverticulum proximal to it. Long segment benign stricture (dashed arrow) is also present throughout the neopharynx in this patient that was likely secondary to a post radiation stricture. **d** This patient had a history of laryngeal squamous cell carcinoma and underwent a total laryngectomy with free flap reconstruction. A TEP was placed for voice restoration (white arrow), and the patient subsequently complained of coughing upon swallowing. Black dashed arrow shows leakage of barium through the TEP resulting in tracheal aspiration

Squamous cell carcinomas (SCC) comprise the vast majority of primary malignancies in the head and neck and may arise from the supraglottic larynx (extending from the epiglottis to the true vocal cords), glottis (true vocal cords), or subglottis (extending from the inferior surface of the true vocal cords to the cricoid cartilage) [46]. Laryngeal SCC most commonly arises in the supraglottis or the glottis region and less commonly in the subglottis (Fig. 5a) [46]. Risk factors include alcohol use, tobacco, and chronic gastroesophageal reflux. Symptoms include dysphagia, hoarseness, odynophagia, otalgia, and bleeding. The imaging appearance of these tumors vary from exophytic, nodular surface irregularity for well-differentiated tumors, to ulceration of the pharyngeal wall which can occur with poorly differentiated tumors [46]. Treatment of SCC involves a multidisciplinary approach consisting of chemotherapy, radiation, and surgery. Early-stage cancers are amenable to vocal-cord sparing surgery and other conversation laryngeal surgeries.

For locally advanced cases, a total laryngectomy may be performed, which involves resection of the hyoid bone, cricoid and thyroid cartilages, vocal folds and epiglottis, with closure and reconstruction of the anterior defect with muscle and skin [34, 47]. On MBSS, the neopharynx appears as a featureless tube extending from the base of the tongue to the cervical esophagus [34]. Complications can occur with total laryngectomy cases such as benign strictures, malignant strictures (Fig. 5b), and pharyngocutaneous fistulas. Following total laryngectomy, a “pseudoepiglottis” may form as a result of scar tissue developing at the tongue base that may enlarge over time due to the pull of the tongue and the pharyngeal constrictors as the patient swallows (Fig. 5c) [48]. This may narrow the entrance to the neopharynx and obstruct transit of the bolus, leading to formation a diverticulum [47, 48].

After total laryngectomy, one of the major challenges a patient encounter is loss of voice. Patients may undergo voice rehabilitation, with placement of a tracheoesophageal prosthesis. The tracheoesophageal prosthesis (TEP) is a one-way valve device which is positioned between the trachea and esophagus. When the patient covers the tracheostomy and exhales, air is forced through the TEP through the esophagus which then vibrates and produces sound. Typically, there is a low rate of complications, but occasionally complications do occur. If the TEP is too small, it could cause leakage around the prosthesis (Fig. 5d). If it is too large, then it may cause obstruction of the esophagus and impairment in phonation. The TEP may dislodge and can be aspirated.

## Esophageal sweep

The interrelationship between the pharynx and the esophagus has long been recognized, and oroharyngeal dysphagia may be caused by esophageal dysfunction [28, 49]. The oral, pharyngeal, and esophageal aspects of the swallowing continuum are interconnected such that dysfunction in one aspect may lead to adaptive or compensatory changes in the other [49, 50]. Multiple studies have shown that patients may identify the pharynx as the site of dysphagia when the abnormality is in the esophagus [51–54]. One study showed that one third of patients that presented with oropharyngeal complaints had abnormal esophageal clearance and would risk being sent home with no diagnosis if the esophageal sweep had not been performed [55]. Therefore, evaluation of swallowing with the MBSS may be incomplete without visualization of the esophagus [56].

Routine visualization of the esophagus during the MBSS has been a matter of debate among SLPs and radiologists, but literature supports the assessment of esophageal clearance on the MBSS and shows that esophageal abnormalities are commonly detected (average of 48.67%) when visualization of the esophagus is included in the MBSS [56]. In a study that evaluated the utility of esophageal visualization using a single 20-mL bolus swallow compared to a full esophagram, the sensitivity was 63% and the specificity was 100% for detecting an esophageal abnormality [57]. Another study showed significant associations between abnormal esophageal clearance on MBSS and abnormal findings on high resolution manometry [28]. Esophageal visualization and emptying assessment can be a simple and important step that impacts appropriate referral for the patient [56, 57]. If a MBSS is performed without the evaluation of the esophagus, the etiology of the oropharyngeal dysphagia may go undiagnosed, which may lead to inaccurate or delayed diagnoses, ineffective treatments, and breakdown in communication among members of the health care team [56]. The division between the oropharynx in the MBSS and the esophagus in the barium swallow is arbitrary and artificial. Assessment of esophageal clearance during the MBSS can provide critical information to allow for appropriate referrals.

Radiologists may be hesitant to follow the bolus through the gastroesophageal junction due to fear of missed pathology. Radiology reports should be clear to describe the limited nature of the esophageal visualization. It is not intended to be a thorough diagnostic assessment of the esophagus, but rather one step to better evaluate the swallowing continuum and better direct management of the patient [56]. Visualization of esophageal clearance in the upright position is also included in the MBSImP which is again a standardized, reliable, and validated protocol for performing the MBSS [24]. The MBPImP includes assessment of the bolus as it passes through the esophagus and the lower esophageal sphincter while the patient is in the upright position [24]. Depending on the findings, full evaluation with an esophagram or appropriate referral would be needed.

## Conclusion

Collaboration between the radiologist and SLP assures optimized performance of the MBSS and the best patient care. The MBSS may be viewed by radiologists as time consuming with low work relative value units (wRVU), which leads to some practices unfortunately prioritizing the radiologist in the reading room interpreting studies with higher wRVUs [8]. There is a misconception that fluoroscopic exams are “low tech” exams and labor intensive [58]. However, the use of MBSS for evaluation of the oropharyngeal swallow continues to increase and radiologists are still needed to be engaged in this exam [2]. Ideally, the radiologist and SLP will discuss the patient before and after the exam to review indications, symptoms, findings, and next steps. Interdisciplinary conferences provide opportunities to a team approach in complex patients. Collaboration of radiologists and speech language pathologists is necessary to provide the best patient care and ensure the best outcomes for the patient.

## Supplementary Information

Below is the link to the electronic supplementary material.

Supplementary file1 (PNG 1005 KB)

## Data Availability

No datasets were generated or analysed during the current study.
