# Supplementary figures and images for: An update on pharyngeal assessment by the modified barium swallow
Source: Abdom Radiol (NY). 2024 Dec 8;50(6):2414–25. doi: 10.1007/s00261-024-04707-9 (PMC12069153; doi:10.1007/s00261-024-04707-9)

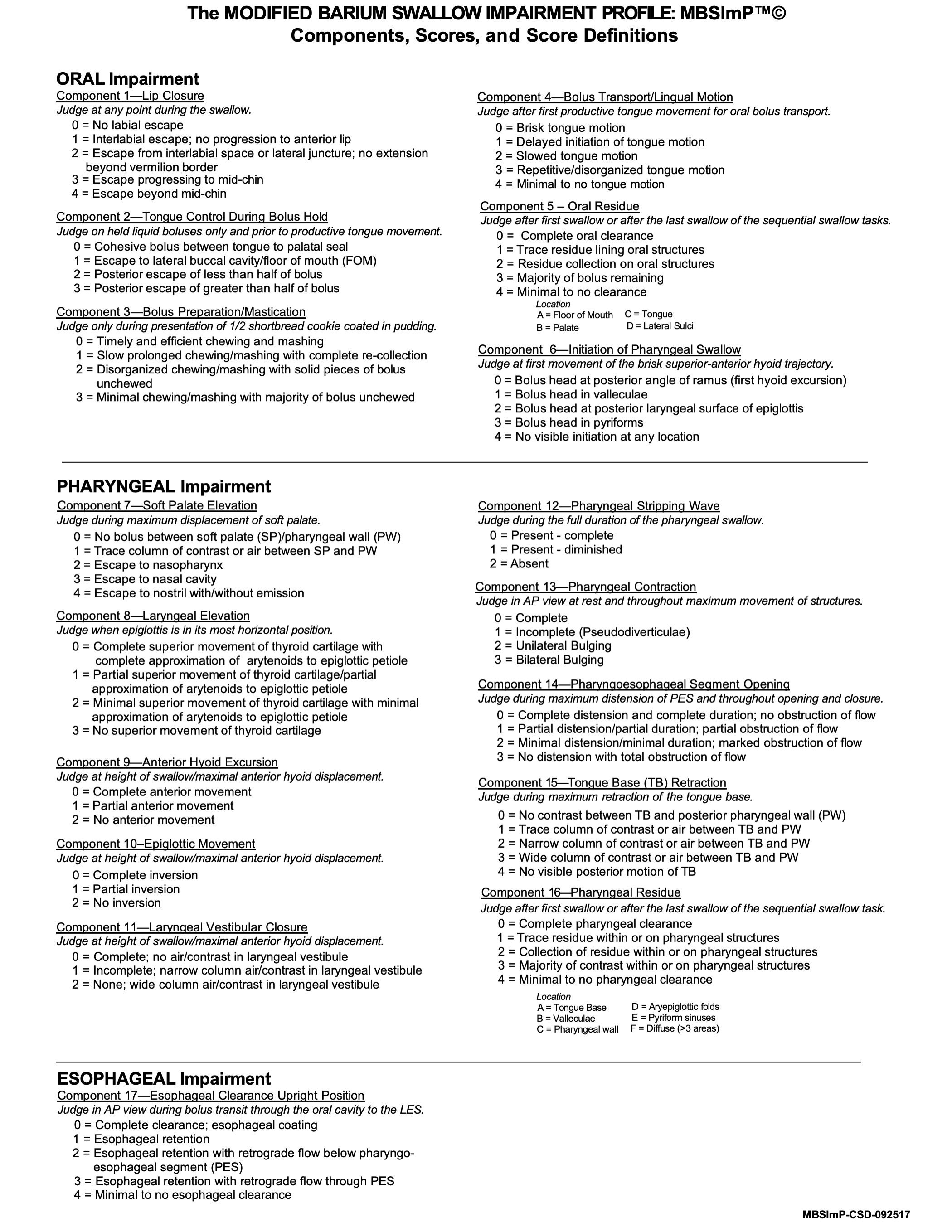

Supplement: Supplementary file 1 — Supplementary file1 (PNG 1005 KB) [file 261_2024_4707_MOESM1_ESM.png]
